# Heterologous Immunity Triggered by a Single, Latent Virus in *Mus musculus*: Combined Costimulation- and Adhesion- Blockade Decrease Rejection

**DOI:** 10.1371/journal.pone.0071221

**Published:** 2013-08-05

**Authors:** Jonathan M. Beus, Salila S. Hashmi, Saranya A. Selvaraj, Danxia Duan, Linda L. Stempora, Stephanie A. Monday, Jennifer A. Cheeseman, Kelly M. Hamby, Samuel H. Speck, Christian P. Larsen, Allan D. Kirk, Leslie S. Kean

**Affiliations:** 1 Emory Transplant Center, Emory University School of Medicine, Atlanta, Georgia, United States of America; 2 Emory Vaccine Center, Emory University School of Medicine, Atlanta, Georgia, United States of America; 3 Department of Microbiology and Immunology, Emory University School of Medicine, Atlanta, Georgia, United States of America; 4 Department of Pediatrics and Children’s Healthcare of Atlanta, Emory University School of Medicine, Atlanta, Georgia, United States of America; New York University, United States of America

## Abstract

The mechanisms underlying latent-virus-mediated heterologous immunity, and subsequent transplant rejection, especially in the setting of T cell costimulation blockade, remain undetermined. To address this, we have utilized MHV68 to develop a rodent model of latent virus-induced heterologous alloimmunity. MHV68 infection was correlated with multimodal immune deviation, which included increased secretion of CXCL9 and CXCL10, and with the expansion of a CD8^dim^ T cell population. CD8^dim^ T cells exhibited decreased expression of multiple costimulation molecules and increased expression of two adhesion molecules, LFA-1 and VLA-4. In the setting of MHV68 latency, recipients demonstrated accelerated costimulation blockade-resistant rejection of skin allografts compared to non-infected animals (MST 13.5 d in infected animals vs 22 d in non-infected animals, p<.0001). In contrast, the duration of graft acceptance was equivalent between non-infected and infected animals when treated with combined anti-LFA-1/anti-VLA-4 adhesion blockade (MST 24 d for non-infected and 27 d for infected, p = n.s.). The combination of CTLA-4-Ig/anti-CD154-based costimulation blockade+anti-LFA-1/anti-VLA-4-based adhesion blockade led to prolonged graft acceptance in both non-infected and infected cohorts (MST>100 d for both, p<.0001 versus costimulation blockade for either). While in the non-infected cohort, either CTLA-4-Ig or anti-CD154 alone could effectively pair with adhesion blockade to prolong allograft acceptance, in infected animals, the prolonged acceptance of skin grafts could only be recapitulated when anti-LFA-1 and anti-VLA-4 antibodies were combined with anti-CD154 (without CTLA-4-Ig, MST>100 d). Graft acceptance was significantly impaired when CTLA-4-Ig alone (no anti-CD154) was combined with adhesion blockade (MST 41 d). These results suggest that in the setting of MHV68 infection, synergy occurs predominantly between adhesion pathways and CD154-based costimulation, and that combined targeting of both pathways may be required to overcome the increased risk of rejection that occurs in the setting of latent-virus-mediated immune deviation.

## Introduction

Solid organ transplantation can enhance quality of life, improve clinical outcomes, and even be life-saving for many individuals with organ failure. Unfortunately, despite treatment with multiple, broadly active immunosuppressive therapies, organ rejection remains a continuous threat for recipients. A growing body of evidence suggests that the immune response to a transplanted organ is significantly influenced by ongoing exposures to environmental antigens. ‘Heterologous alloimmunity’ is a multimodal phenomenon wherein continual encounters of the immune system with stimuli such as viral antigens affect the immune repertoire, alter immunosuppressive requirements, and lead to breakthrough rejection in several experimental models [1]–[4]. Although heterologous immunity is commonly attributed to TCR-cross-reactivity, evidence suggests that additional features of immune activation such as altered cytokine production, bystander activation, or alteration of the T-helper 1 to T-helper 2 balance may contribute to such an effect [4], [5].

While the majority of studies of virus-induced heterologous immunity have employed either acute viral infection models [1], [6], [7], or transgenic systems which predominantly model single-antigen receptor cross-reactivity [8], the most relevant clinical scenario is one of viral latency, with the most prominent clinical manifestations occurring from two latent viruses: CMV and EBV. To accurately recapitulate this clinical scenario, we previously established a model of latent-virus mediated heterologous alloreactivity using the EBV homolog [9]–[12] MHV68, and demonstrated that infection with this single latent virus can profoundly influence bone marrow transplant rejection and tolerance-induction [3].

The emerging role of T cell costimulation blockade (CoB)-based immunosuppression regimens in solid organ transplantation has drawn increased attention to heterologous alloimmunity. This is due to the large body of work that has implicated memory T cells (T_M_, often expanded during the heterologous immune response [1], [5]) as being particularly costimulation blockade resistant [1], [13], [14]. Indeed, while CD28-directed CoB with belatacept, recently FDA approved, in renal transplant recipients resulted in better long-term renal function [15], [16], CoB-treated patients experienced more acute rejection than those on standard calcineurin inhibitor-based therapy [16]. This raises the concern that patients who develop a T_M_-skewed immune repertoire in the face of latent viral infection may be at risk for costimulation blockade-resistant rejection (CoBRR). Thus, there is a critical need to understand the mechanisms by which virus-mediated heterologous alloimmunity impacts allograft rejection and how this rejection response may be overcome. To accomplish this, with particular attention to solid organ allografts, we have now developed a skin graft model in mice latently infected with MHV68 and have determined strategies to overcome the increased risk of rejection that we observed in MHV68-infected transplant recipients.

## Materials and Methods

### Mice and Viral Infections

C57BL/6 and BALB/c mice (6–12 weeks old) were purchased from the Jackson Laboratory (Bar Harbor, ME). Virus stocks were grown and quantitated as previously described [17]. Mice were infected i.p. with 10^5^ plaque-forming units WT or M1.STOP MHV68 [18] and latency was defined as approximately 6 weeks post-infection [17]. Quantitation of absolute virus concentration was performed after extraction of DNA from fixed quantities of whole blood (QIAmp DNA kit, Qiagen, Valencia, CA) using previously described cycling conditions and primer-probe sets for MHV68 ORF50 [19]. All animal studies were approved by the Institutional Animal Care and Use Committee of Emory University (PHS Assurance A3180-01). All surgery was performed under anesthesia with fentanyl, midazolam, and droperidol.

### Skin Grafting and Antibody Administration

Full-thickness skin grafts from donor mice were placed on the dorsal thorax of recipient mice at 6 weeks after infection or on age-matched controls and secured with adhesive bandages. Rejection was declared when less than 10% of epidermal tissue remained viable upon visual inspection.

CoB-treated mice received 500 µg each of hamster anti-mouse CD154 mAb (MR-1, Bio X Cell, West Lebanon, NH) and human CTLA-4-Ig (Abatacept, Bristol-Meyers Squibb, New York, NY) i.p. at transplantation and on post-transplant days 2, 4, and 6. Mice in indicated groups received 250 µg anti-mouse LFA-1 mAb (anti-CD11a mAb, clone M17/4) and/or 250 µg anti-mouse VLA-4 mAb (clone PS/2) i.p. (Bio X Cell) at transplantation, on post-transplant days 2, 4, 6, and weekly thereafter. Appropriate mice received 200 µg of anti-mouse Vβ4 mAb (clone KT4, hybridoma gift of George S. Deepe) or rat IgG2b isotype control (clone LTF-2) (both from Bio X Cell) i.p. 4 and 2 days prior to infection, at infection, and weekly after infection.

Studies of adhesion blockade withdrawal were performed on MVH68 WT- or M1.STOP-infected mice with stable allografts on weekly anti-LFA-1/anti-VLA-4 treatment following CoB treatment. After 90–100 days of treatment, adhesion blockade therapy was discontinued and skin graft viability was monitored as described above.

### Flow Cytometry

Peripheral blood was prepared with fixative-free lysing solution (Invitrogen, Carlsbad, California) and stained with the indicated mAbs at 4°C. Antibodies were used against CD8 (53–6.7, PE), Vβ4 (KT4, FITC), CD44 (IM7, V500), and 4-1BB (1AH2, FITC) from BD Biosciences (Franklin Lakes, NJ), CD3 (17A2, eFluor 450), CD127 (A7R34, Alex Fluor 700), KLRG1 (2F1, PE-Cy7), and VLA-4 (R1-2, FITC) from eBioscience (San Diego, CA), and bcl2 (BCL/10C4, Alex Fluor 647), CD28 (37.51, PerCP-Cy5.5), ICOS (15F9, PE-Cy5), and CD11a (M17/4, Alexa Fluor 647) from Biolegend (San Diego, CA). BD Biosciences TruCOUNT tubes were used to determine absolute cell counts. Antigen-specific staining was performed against MHV68 p56 (AGPHNDMEI) and p79 (TSINFVKI) antigens [20], [21] with class I tetramers (NIH Tetramer Core Facility, Emory University, Atlanta, GA). Intracellular staining for IFN-γ was performed on peripheral blood cells stimulated with 1 µg/mL of each p56 and p79 (GenScript, Piscataway, NJ) using the BD Biosciences Fixation/Permeabilization Solution Kit with brefeldin A. All samples were acquired on a BD Biosciences LSRII flow cytometer and analyzed using FlowJo (Tree Star, Ashland, OR). Some flow cytometry plots, as noted in their respective legends, represent concatenation of equal numbers of cells from multiple animals to demonstrate that similar phenotypic distributions were observed in biological replicates.

### Cytokine Assay

Peripheral blood collected prior to infection and every two weeks after infection was centrifuged for 10 min at 1000 G to separate plasma. Samples were frozen at −80°C until assayed using the premixed 32-plex or a custom MILLIPLEX MAP Mouse Cytokine/Chemokine kit (Millipore, Billerica, MA) on a Bio-Plex 200 system (Bio-Rad, Hercules, CA).

### Cell Sorting and Gene Expression Analysis

Suspensions of splenocytes from mice 4–8 weeks after infection or from age-matched controls were prepared by grinding each spleen through a 40 µm cell strainer. The cell suspensions from multiple mice were combined to increase RNA yield in some experiments. Following treatment with fixative-free lysing solution (Invitrogen), cells were stained with mAbs to CD8 (53–6.7, PE), CD19 (1D3, APC), NK1.1 (PK136, APC) from BD and CD4 (RM4-5, APC, Invitrogen). On a BD FACSAria, CD8+/CD4−/CD19−/NK1.1- cells were sorted from non-infected animals or additionally sorted into CD8^bright^ and CD8^dim^ for cells from infected animals. RNA was then extracted using the Qiagen RNeasy kit with DNase treatment prior to reverse transcription to cDNA using an AMV First Strand cDNA Synthesis Kit (Roche Diagnostics, Indianapolis, IN) or a High Capacity cDNA Reverse Transcription Kit (Applied Biosystems, Carlsbad, CA). Applied Biosystems TaqMan Mouse Immune Arrays (V2.1) were run and acquired with an Applied Biosystems 7900HT Fast Real-Time PCR System. Data were analyzed with SAS 9.2 (SAS Institute, Cary, NC) using the ΔΔCt method with normalization to β-actin. The analysis included cDNA from cells of 4 groups of non-infected mice, CD8^dim^ cells from 6 groups of infected mice, and CD8^bright^ cells from 7 groups of infected mice from 3 individual experiments (1–4 technical replicates per measurement).

### Statistical Analysis

Statistical analyses were performed with GraphPad Prism 5 (La Jolla, CA), SAS 9.2, and R 2.12 or greater (Vienna, Austria). Skin graft survival between groups was compared with the log-rank test. Kruskal-Wallis tests with Dunn’s multiple comparison test for post-hoc pairwise comparisons were used in analyses of cytokine concentration and viral load. For analysis of gene expression between cell types, the Kruskal-Wallis test was used to compare across all cell types and the false-discovery rate method was used to correct for repeated testing across multiple analytes. Among analytes with significant differences across all cell types, pairwise comparisons between cell types were made using Mann-Whitney U tests with Bonferroni corrections for multiple pairwise comparisons. Tests were performed at the 5% significance level.

## Results

### Latent MHV68 Infection Accelerates Skin Allograft Rejection

**Figure 1:**
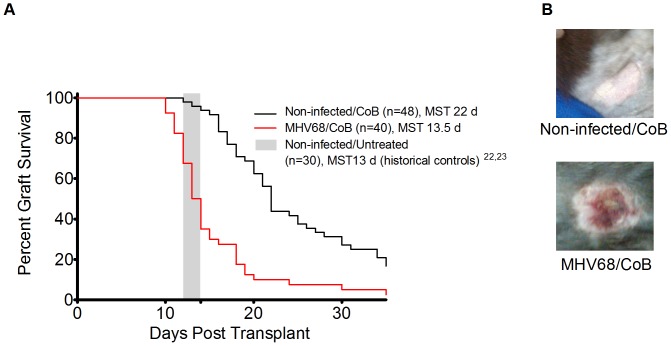
Latent infection with MHV68 results in abbreviated skin allograft survival. A) Kaplan-Meier survival curves showing skin graft survival for the following cohorts: Black solid line - Non-infected B6 recipients of BALB/C skin allografts, receiving CTLA-4-Ig+anti-CD154 CoB. N = 48, 8 independent experiments, MST 22 d. Red solid line - MHV68-infected B6 recipients of BALB/c skin allografts receiving CTLA-4-Ig+anti-CD154 CoB. N = 40, 7 independent experiments, MST 13.5 d. Grey box - indicates MST from two historical control cohorts [22], [23] of non-infected B6 recipients receiving BALB/c skin grafts without any immunosuppression (MST 13 d). Statistical Analysis: Log-rank comparison of MHV68-infected/CoB treated versus non-infected/CoB treated yielded p<.0001. Log-rank comparison of MHV68-infected/CoB treated to non-infected/no immunosuppression yielded a non-significant p-value. B) Representative skin allografts from mice 12 days after graft placement. The graft on the non-infected animal remains healthy and pristine while the graft on the animal infected with latent MHV68 demonstrates necrosis, scarring, and erythema.

Skin graft survival in the setting of CoB treatment (combined CTLA-4-Ig and anti-CD154) was compared between MHV68-infected and non-infected mice to determine whether latent MHV68 augmented the alloimmune response to skin grafts in the stringent BALB/c to B6 system. As shown in Figure 1, while BALB/c skin grafts on non-infected, CoB-treated B6 mice exhibited a median survival time (MST) of 22 d (n = 48 from 8 independent experiments, p<.0001 compared to the previously-reported [22], [23] MST of 13 d for non-infected animals receiving no immunosuppression, n = 30). As shown in the figure, latent infection with MHV68 significantly decreased the MST in CoB-treated recipients to 13.5 d (n = 40 from 7 independent experiments, p<.0001 compared to CoB-treated non-infected recipients and p = n.s. compared to non-infected historical controls receiving no immunosuppression) [22], [23]. Given the short allograft survival time in MHV68-infected animals treated with CoB, the comparator group of infected animals not treated with CoB was not included in this study. Of note, all syngeneic (B6) grafts on MHV68-infected B6 mice survived for greater than 100 d (n = 6, not shown), confirming that the impact of MHV68 latency was on the alloimmune response, and did not impair the secondary vascularization that must occur for either syngeneic or allogeneic skin graft survival. These data demonstrate that even in the immunologically stringent BALB/c to B6 skin graft model, latent virus-mediated alloimmune effects occur and are measurable clinically in a highly reproducible fashion.

### Phenotypic and Functional Immune Alteration After MHV68 Infection

To determine the effect of MHV68 latency on the immune phenotype, we undertook an analysis of cytokine- and T cell-mediated immunity in latently-infected animals, including flow cytometric analysis of peripheral blood T cells, quantitation of plasma cytokines and chemokines, and quantitative PCR array-based immune gene expression analysis of sorted CD8+ T cells.

**Figure 2:**
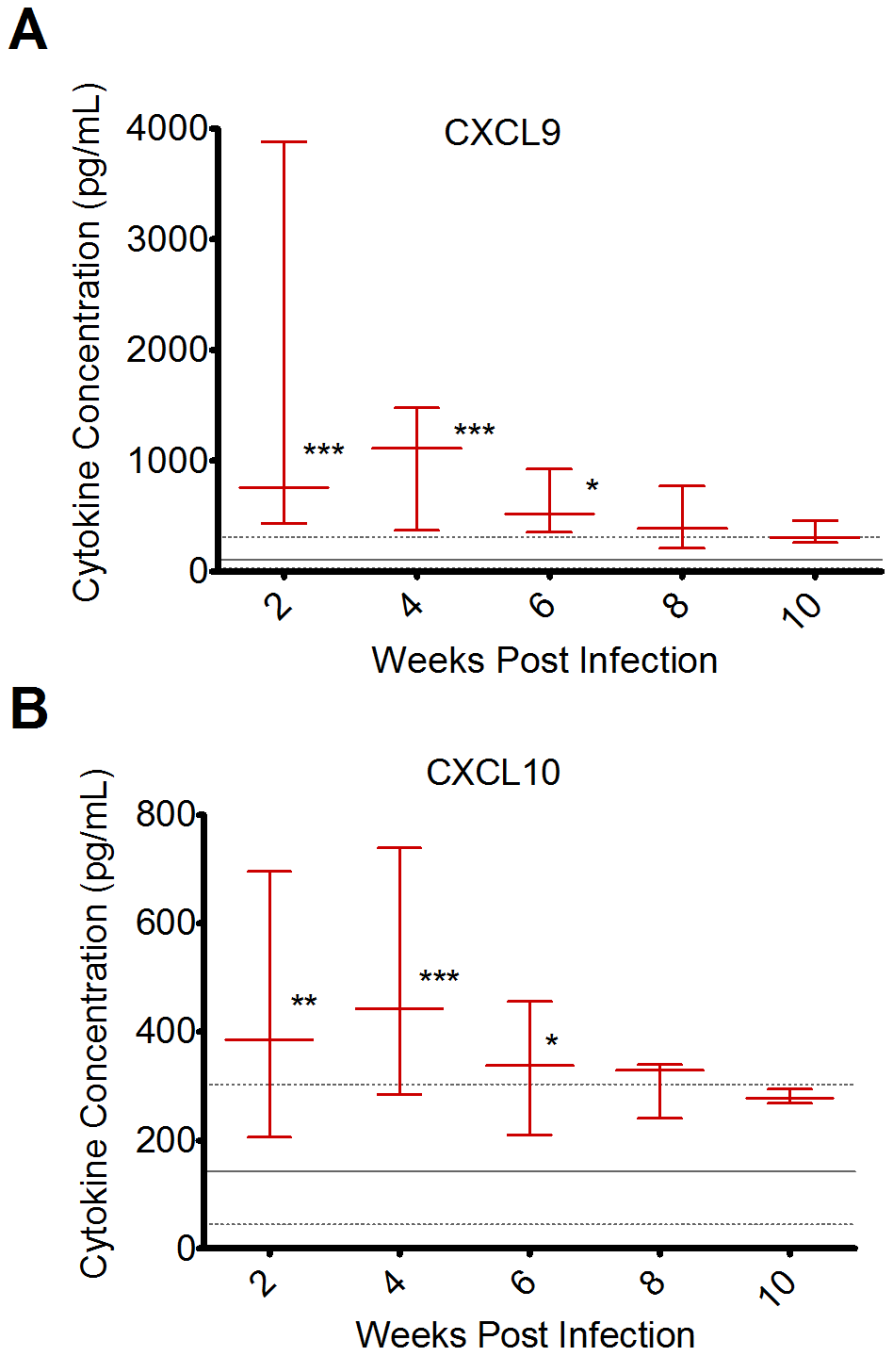
MHV68 causes systemic elevation of the chemoattractant chemokines CXCL9 and CXCL10. A) Quantification of CXCL9 in the plasma of MHV68-infected animals measured every 2 weeks for 10 weeks after infection. B) Quantification of CXCL10 in the plasma of MHV68-infected animals measured every 2 weeks for 10 weeks after infection. For both (A) and (B) red lines and error bars indicate medians and ranges, respectively, for MHV68-infected animals. Non-infected animals are represented by solid gray lines (medians) and dashed gray lines (ranges). Each point represents 3-7 mice with 1-3 technical replicates each. Levels for MHV68-infected animals at each timepoint were compared to non-infected animals using the non-parametric Kruskal-Wallis test with Dunn’s multiple comparison test. *p<.05, **p<.01, ***p<.001.

The plasma levels of 32 cytokines and chemokines (listed in Table S1) were assessed approximately every two weeks after infection for 10 weeks and compared to levels in non-infected animals. Many analytes (listed in Table S1) where either undetectable in the plasma or exhibited similar levels for both infected and non-infected animals (not shown). In contrast, significant increases in the levels of the chemokines CXCL9 (MIG) and CXCL10 (IP-10), previously demonstrated to play key roles in the trafficking of lymphocytes to sites of inflammation [24], [25] and known to be associated with clinical allograft rejection [26], [27], were observed in infected animals relative to non-infected mice at 2, 4, and 6 weeks after infection (Figure 2). As shown in Figure 2, the median CXCL9 concentration in the serum increased from 105 pg/mL to 755 pg/mL, 1110 pg/mL, and 521 pg/mL at 2, 4, and 6 weeks post-infection, respectively (p<.001, <.001, and <.05, respectively, compared to non-infected controls). Similarly, the median CXCL10 concentration in the serum increased from 142 pg/mL to 385 pg/mL, 443 pg/mL, and 338 pg/mL at 2, 4, and 6 weeks post-infection, respectively (p<.01, <.001, and <.05, respectively, compared to non-infected controls).

**Figure 3:**
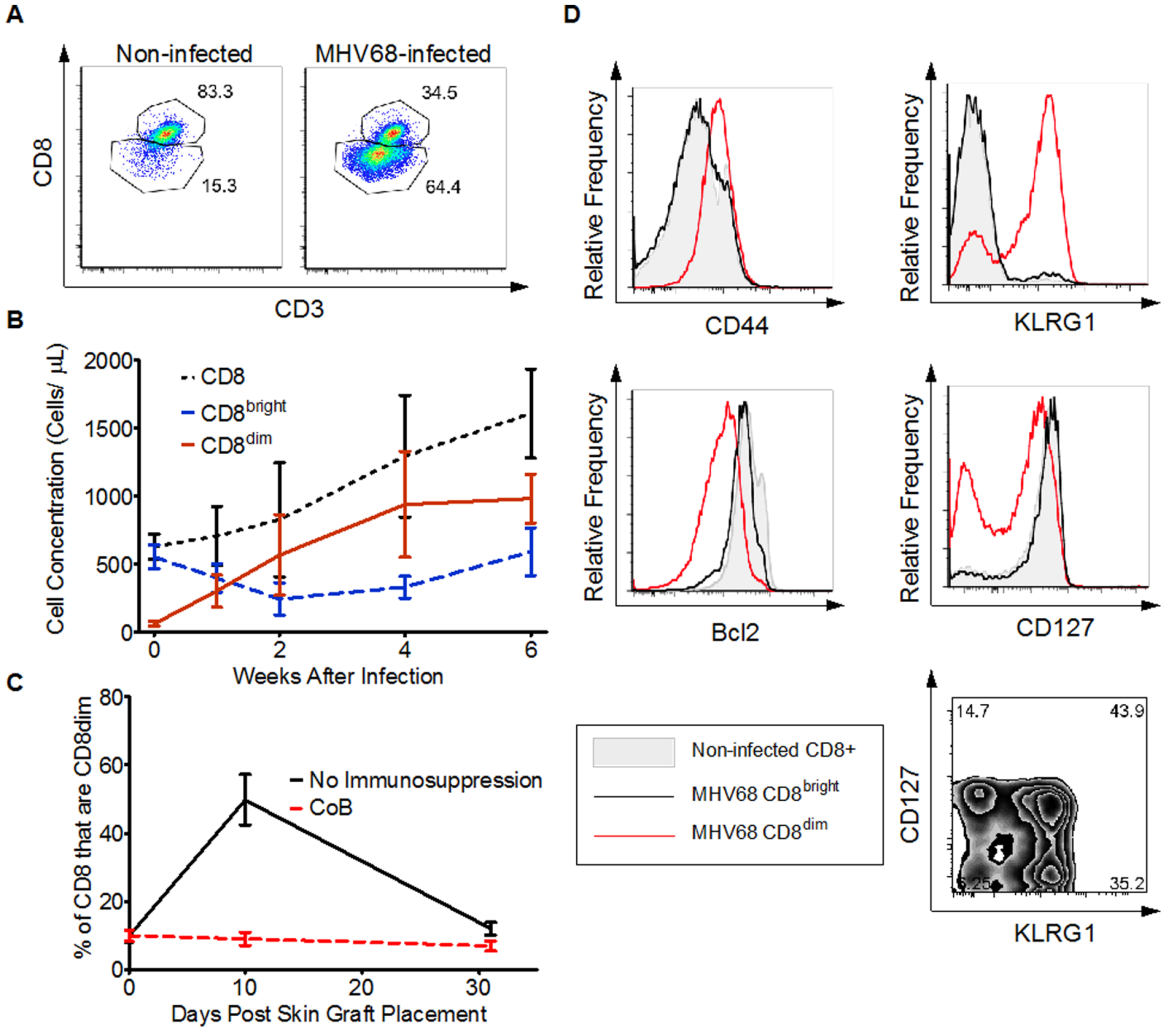
MHV68 results in the expansion of a unique T cell population, CD8^dim^. A) Flow cyotmetric plots showing CD8 versus CD3 staining after gating on lymphocytes by forward and side scatter followed by gating on CD3+/CD8+ cells. Flow cytometry was performed on peripheral blood samples drawn 6 weeks post-infection or in similarly aged non-infected animals. Each plot shows the combined results from equal numbers of cells from 5 animals. B) Longitudinal analysis in MHV68-infected animals of the absolute numbers of total CD8 (dotted black line), CD8^bright^ (dashed blue line), and CD8^dim^ (solid red line) T cells following infection. Data include 10 mice from 2 independent experiments except at 1 week where 5 mice from a single experiment are displayed. Error bars represent 1 standard deviation. C) Longitudinal analysis in non-infected B6 mice of the percentage of CD8 T cells that are CD8^dim^ after placement of allogeneic skin grafts. The solid black line represents mice not receiving immunosuppressive therapy (n = 4). The dashed gray line shows mice receiving CTLA-4-Ig+anti-CD154 therapy (n = 6). Error bars show 1 standard deviation. D) Flow cytometric analysis at 6 weeks after infection comparing the relative expression of CD44, KLRG1, Bcl2, and CD127 with histograms between CD8 T cells from non-infected animals (gray filled area), CD8^bright^ (solid black line) from MHV68-infected animals, and CD8^dim^ (solid red line) from MHV68-infected animals. The plot in the bottom right shows the expression of CD127 versus KLRG1 for CD8^dim^ in MHV68-infected animals. Each cell type for each plot shows the combination of 5 individual mice.

Flow cytometric analyses were performed longitudinally after infection to examine T cell phenotypic changes that correlated with virus-induced alloimmunity. Within 2 weeks of infection, we observed a large expansion of a distinct T cell population with decreased cell surface expression of both CD8 and CD3 (“CD8^dim^”, Figures 3A and 3B). While only approximately 15% of CD3+CD8+ cells were CD8^dim^ in non-infected mice, CD8^dim^ cells comprised more than 60% of CD8+ T cells in MHV68-infected mice at 6 weeks after infection (Figure 3A). As late as 165 days after infection, we observed that 63% of CD3+CD8+ cells were CD8^dim^ (n = 4, not shown), demonstrating that this cell population persists long after the acute phase of the viral infection. The correlation of decreased CD8 expression with the propensity towards rejection was suggested by the fact that a similar population of CD8^dim^ cells transiently expanded in non-infected animals undergoing skin allograft rejection (Figure 3C).

While technical barriers precluded adoptive transfer experiments to investigate a causal relationship between CD8^dim^ expansion and transplant rejection, the correlation of their expansion with virus-induced rejection prompted a more thorough examination of the phenotype of these cells. In agreement with the expansion of CD8^dim^ after viral infection, the CD8^dim^ subpopulation exhibited characteristics of effector and effector memory (T_EM_) T cells. Relative to both CD8+ T cells from non-infected animals and to the CD8^bright^ T cells in MHV68-infected animals, CD8^dim^ had nearly universal increased expression of the activation markers CD44 and KLRG1, and decreased expression of the anti-apoptotic molecule bcl2 (Figure 3D). As shown in Figure 3D, over 40% of CD8^dim^ simultaneously expressed KLRG1 and CD127, suggesting that a significant fraction of CD8^dim^ were T_EM_ [28].

### CD8^dim^ Exhibit Downregulation of Cell Surface Costimulation Molecules and Significant Upregulation of Cell Surface Adhesion Molecules

**Figure 4:**
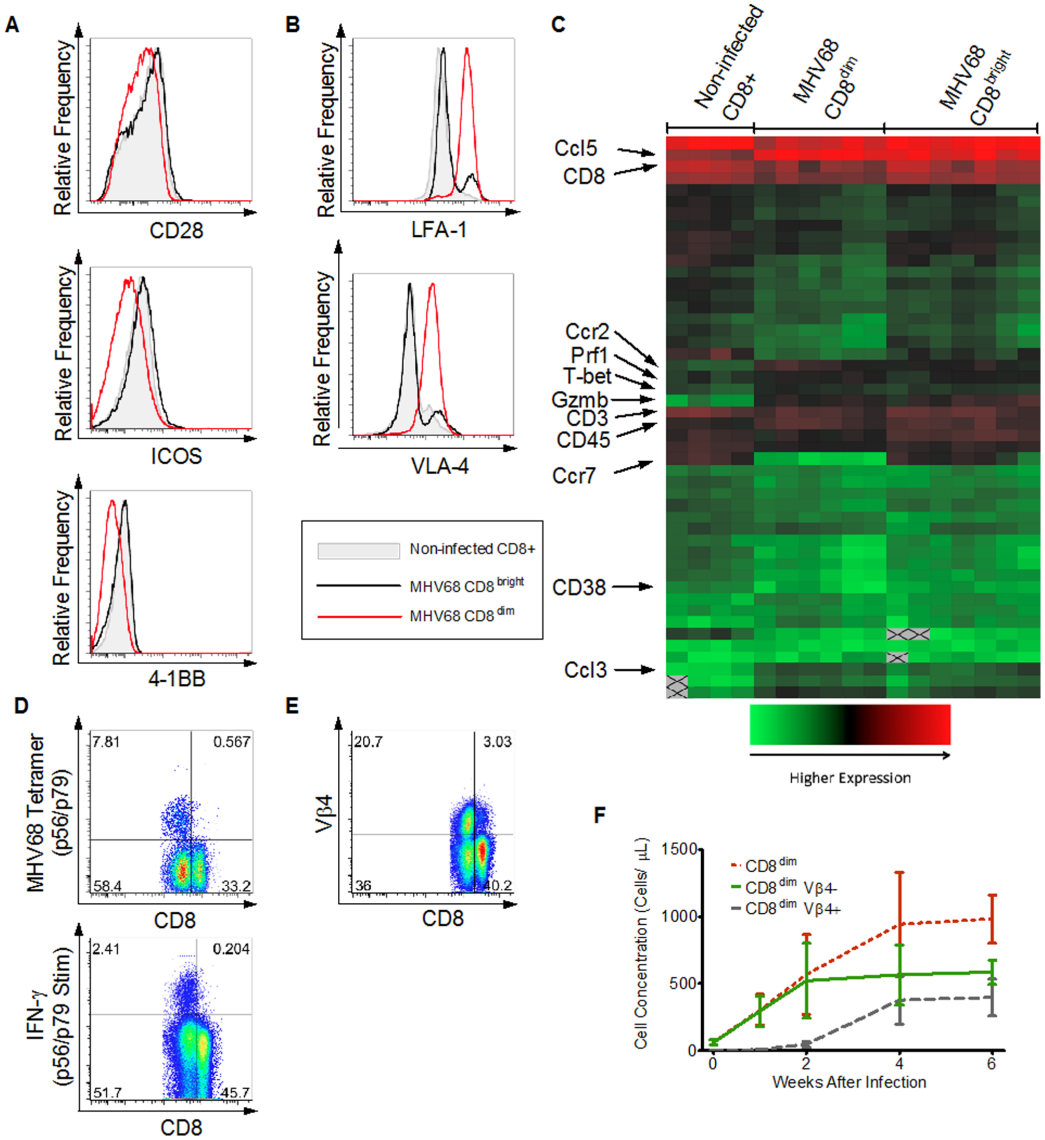
CD8^dim^ exhibit decreased expression of costimulatory molecules and increased expression of adhesion molecules. A) Histograms from flow-cytometry based-phenotyping at 6 weeks after infection (or age-matched, non-infected controls) compare the relative expression of the costimulatory molecules CD28, ICOS, and 4-1BB between CD8 T cells from non-infected animals (gray filled area), CD8^bright^ (solid black line) from MHV68-infected animals, and CD8^dim^ (solid red line) from MHV68-infected animals. Each cell population represents the combined results from 5 individual mice. Similar results (not shown) were observed 3 weeks after infection. B) Flow-cytometric phenotyping from mice 6 weeks after infection (or age-matched, non-infected controls) compare the relative expression of the adhesion molecules LFA-1 and VLA-4 between CD8 T cells from non-infected animals (gray filled area), CD8^bright^ (solid black line) from MHV68-infected animals, and CD8^dim^ (solid red line) from MHV68-infected animals. Each cell population represents the combined results from 5 individual mice. Similar results (not shown) were observed 3 weeks after infection. C) Comparison of gene expression profiles between CD8+ T cells from non-infected animals, CD8^bright^ from infected animals, and CD8^dim^ from infected animals (additional data in Table S2). Data shown from 3 independent experiments, with 1–3 biologic replicates and 1–4 technical replicates each. D) Flow cytometric analysis of CD8+ T cells from MHV68-infected animals at 6 weeks after infection identifies MHV68-specific CD8 T cells by the use of both MHC tetramers and peptide-stimulated IFN-γ release. The plots show MHV68-specificity versus CD8 surface expression. Each plot represents combined results from 5 mice. E) Flow cytometric analysis of CD8+ T cells from MHV68-infected mice at 6 weeks after infection demonstrating TCR Vβ4 staining versus CD8 staining. Each plot represents combined results from 5 mice. F) Longitudinal analysis of total CD8^dim^ cells (dotted red line), CD8^dim^ Vβ4- (solid green line), and CD8^dim^ Vβ4+ (dashed gray line) from MHV68-infected mice 6 weeks after infection (n = 10, except for week 1; n = 5). Error bars represent 1 standard deviation.

Given that CD8^dim^ cells expressed phenotypic markers of T_EM_, we next determined the expression pattern of costimulation and adhesion molecules on these cells [14], [29], [30]. As shown in Figure 4A, flow cytometric analysis revealed decreased expression of the costimulatory molecules CD28, ICOS, and 4-1BB relative to CD8^bright^ in infected animals and to total CD8+ cells from non-infected animals. We also observed significantly increased expression of the adhesion molecules LFA-1 and VLA-4 on the majority of CD8^dim^ (Figure 4B).

To further characterize the effects of MHV68 infection on the CD8+ T cell phenotype, CD8^bright^ and CD8^dim^ T cells were individually isolated using a FACSAria flow sorter from splenocytes of infected animals by identification and subsequent purification of CD8^bright or dim^/CD4−/CD19−/NK1.1- cells and compared to CD8+/CD4−/CD19−/NK1.1− cells purified from non-infected animals. RNA was isolated from the purified cells and mRNA transcripts in these populations were compared using quantitative PCR arrays. As shown in Figure 4C and Table S2, two comparisons were made: CD8^dim^ and CD8^bright^ T cells from infected animals were compared to each other and also to total CD8+ cells from non-infected animals. Relative to CD8+ cells from non-infected animals, CD8+ T cells from infected animals (both CD8^dim^ and CD8^bright^) exhibited significant (>2 fold difference in expression at the 5% significance level after applying the Bonferroni correction for pairwise comparisons) differential expression of 9 of 50 genes tested (Table S2). As shown in Figure 4C and Table S2, several notable variations in expression levels among these included upregulation of the chemoattractants CCL3, CCL5 (RANTES) [31], upregulation of CCR2 (receptor for the chemoattractant MCP-1) [32], upregulation of CD38 (associated with activation of T cells) [33], upregulation of several cytotoxicity proteins (granzyme-B and perforin 1) [34], upregulation of the T cell signaling molecule Ptprc (CD45) [35], upregulation of the IFN-γ transcription factor Tbx21 (T-bet) [36], and downregulaton of the anti-apoptotic enzyme HMOX2 [37]. Comparison of CD8^dim^ to CD8^bright^ in infected animals also revealed differential expression of several genes. As shown in Figure 4C and Table S2, this included increased transcription of the chemoattractant chemokine CCL5 [31] and the chemokine receptor CCR2 [32] in CD8^dim^, but lower transcription of CCR7 (important in homing T cells to lymph nodes) [38] and Stat1 (part of the IFN-γ signaling pathway) [39]. In contrast, the relative transcription of CD3 and CD8 in CD8^dim^ vs CD8^bright^ (±1 S.D.) was found to be 1.07 (0.64–1.78) for CD3 and 0.86 (0.72–1.02) for CD8, suggesting that the differences observed in cell surface expression of these proteins were due primarily to post-transcriptional effects.

To determine the relationship between the CD8^dim^ expansion and anti-viral T cells, two immunodominant H-2^b^-restricted MHV68 epitopes (p56 and p79) [21], [40] were used to identify viral antigen-specific T cells at approximately 6 weeks post infection by both tetramer staining and peptide-stimulated IFN-γ production. As shown in Figure 4D, using either technique, nearly all anti-viral cells were CD8^dim^, suggesting that CD8^dim^ expansion is at least partly driven by viral-specific T cell proliferation (Figure 4D). While the majority of Vβ4+CD8+ cells (previously shown to expand after MHV68 infection [11], [41]), were CD8^dim^ (Figure 4E), most CD8^dim^ expansion in the first 2 weeks after infection occurred in Vβ4-negative CD8+ T cells (Figure 4F). Moreover, even after the peak of Vβ4+CD8+ expansion between 2–6 weeks post-infection, Vβ4-negative CD8+ T cells represented a major fraction of the CD8^dim^ population (Figure 4F). Given that Vβ4+CD8+ expansion is not considered to result from specificity for any MHV68 antigens [18], [42], and that many Vβ4+CD8+ cells do not specifically recognize the dominant p56 and p79 viral epitopes [21], [43] (not shown), the expansion of CD8^dim^ cells is likely the result of both specific (anti-viral) and non-specific immune activation driven by viral infection.

### Skin Graft Rejection in Latent MHV68-infected Mice Occurs in the Absence of M1 Gene Function or Vβ4+CD8+ T cell Expansion

**Figure 5:**
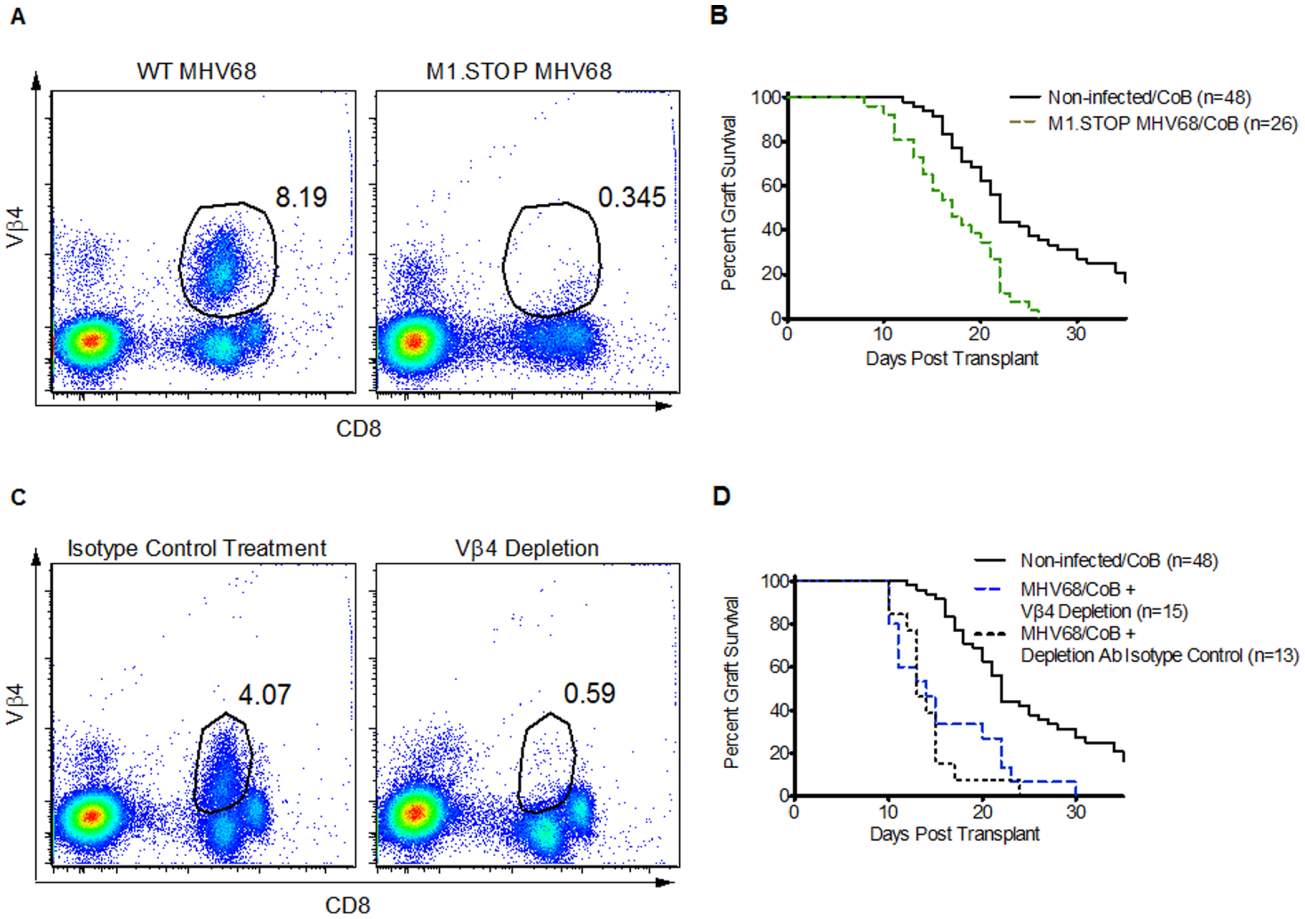
Abbreviated skin allograft survival in MHV68 is not due to MHV68-associated Vβ4+CD8+ expansion. A) Representative flow cytometric plots showing TCR Vβ4 staining versus CD8 staining in mice infected with wild-type MHV68 (left) and the mutant M1.STOP MHV68 (right). B) Kaplan-Meier survival curves comparing skin graft survival for non-infected mice treated with CoB (solid black line, MST 22 d, n = 48, 8 independent experiments) and those infected with the M1.STOP MHV68 mutant virus also treated with CoB (dashed green line, MST 17 d, n = 26 from 3 independent experiments). The log-rank comparison of graft survival between the two groups yielded p<.0001. C) Representative flow cytometric plots showing TCR Vβ4 expression versus CD8 expression in mice infected with wild-type MHV68. The animal in the plot on the left received a non-functional antibody of the same isotype as the antibody used for depletion of Vβ4+ cells (right plot). D) Kaplan-Meier survival curves comparing skin graft survival in non-infected mice treated with CoB (solid black line, MST 22 d, n = 48, 8 independent experiments), MHV68-infected mice treated with a Vβ4-depleting antibody (dashed blue line, MST 14 d, n = 15, 2 independent experiments), and MHV68-infected mice treated with a non-functional antibody (dotted black line, MST 13 d, n = 13, 2 independent experiments) of the same isotype as the Vβ4-depleting antibody. Statistical comparison of the two MHV68-infected cohorts (depleted and isotype control) with the log-rank method yielded a non-significant p-value while comparison of either of the MHV68-infected cohorts with the non-infected cohort yielded p<.0001 despite Vβ4 depletion.

Our previous observations in the bone marrow transplant model implicated both viral M1 gene function and Vβ4+CD8+ T cell expansion in the rejection of allogeneic bone marrow during MHV68 latency [3]. However, as shown in Figure 5, in this skin allograft model, using both an M1-deficient MHV68 mutant virus [18] and antibody-mediated depletion of Vβ4+CD8+ T cells, we observed that M1 gene function is dispensable for MHV68-mediated rejection. Thus, as shown in Figures 5A and 5B, mice infected with the MHV68 M1.STOP mutant virus (which lacks both M1 function and M1-dependent Vβ4+CD8+ T cell expansion) exhibited CoBRR (MST = 17 d, n = 26) relative to non-infected mice (MST = 22 d, p<.0001). We further confirmed that Vβ4+CD8+ T cells were not causative of CoBRR in the skin graft model by antibody-mediated depletion of Vβ4+ cells [44] from infected animals. While Vβ4+ T cells are exceedingly difficult to deplete after virus-mediated expansion [18], we found that Vβ4+ T cells could be effectively depleted when antibody treatment was begun before and continued after MHV68 infection. Depletion was confirmed by the absence of staining for both Vβ4+ (Figure 5C) and the isotype of the antibody (not shown). In agreement with the experiments utilizing the M1.STOP virus, infected mice depleted of Vβ4+ cells still experienced significantly accelerated skin allograft rejection (Figure 5D, MST = 14 d, n = 15) relative to non-infected mice (MST = 22 d, p<.0001). Thus, while bulk Vβ4+CD8+ expansion may be sufficient to drive rejection of bone marrow [3], these cells do not appear to mediate skin allograft rejection. As such, the skin graft model described herein may be able to better model the clinical impact of latent viral infection in patients, in which expansion of a similar TCR-β-chain-restricted T cell subset is not generally observed [45], [46].

### Blocking Adhesion Pathways in Addition to Costimulation Pathways Overcomes MHV68-associated CoBRR

**Figure 6:**
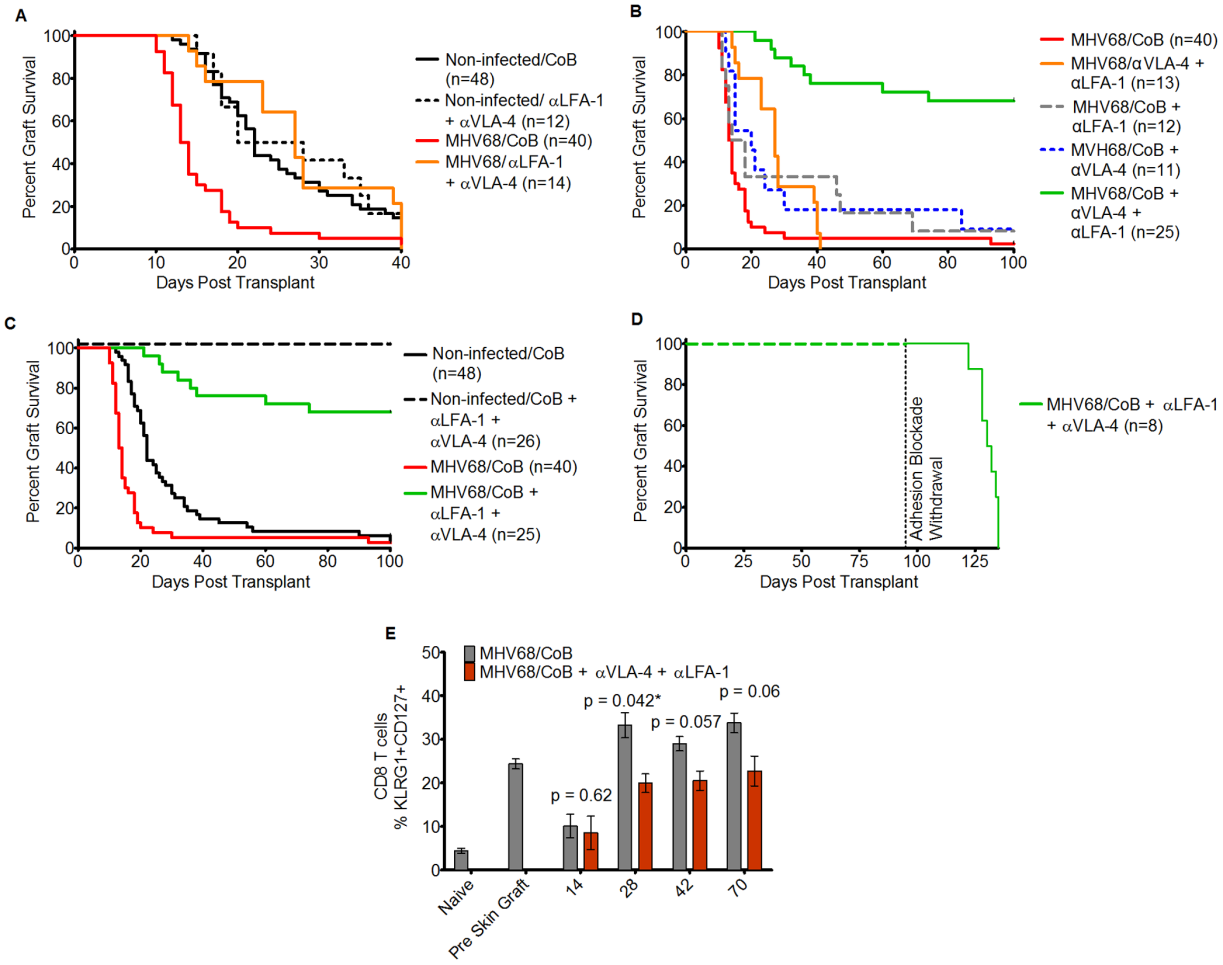
Combined Blockade of costimulation and adhesion pathways overcomes heterologous alloimmunity. A) Kaplan-Meier survival curves comparing skin graft survival between non-infected, CoB treated mice (solid black line, MST 22 d, n = 48, 8 independent experiments), non-infected mice treated with anti-LFA-1 and anti-VLA-4 antibodies (dotted black line, MST 24 d, n = 12, 2 independent experiments), MHV68-infected, CoB treated mice (solid red line, MST 13.5 d, n = 40, 7 independent experiments), and MHV68-infected mice treated with anti-LFA-1 and anti-VLA-4 antibodies (solid orange line, MST 27 d, n = 14, 2 experiments). When non-infected, CoB treated mice were compared to non-infected, anti-LFA-1+ anti-VLA-4 treated mice with the log-rank test, no significant difference was observed (p = n.s.). Comparison of MHV68-infected, anti-LFA-1+ anti-VLA-4 treated mice to non-infected mice treated with either regimen also yielded a non-significant p-value. MHV68-infected, anti-LFA-1+ anti-VLA-4 treated mice demonstrated significantly different skin graft survival compared to MHV68-infected, CoB-treated animals (p = .002). B) Kaplan-Meier survival curves comparing skin graft survival between the following groups: MHV68-infected mice treated with CoB (solid red line, MST 13.5, n = 40, 7 independent experiments); MHV68-infected mice treated with anti-LFA-1/anti-VLA4 (solid orange line, MST 27 d, n = 13, 2 independent experiments); MHV68-infected mice treated with CoB+anti-LFA1 (dotted grey line, MST 16, n = 12, 2 independent experiments); MHV68-infected mice treated with CoB+anti-VLA4 (dotted blue line, MST 20 d, n = 11, 2 independent experiments); MHV68-infected mice treated with CoB+anti-LFA1/anti-VLA4 (solid green line, MST>100 d, n = 25, 3 independent experiments). Statistical comparisons were made with the log-rank test. In infected animals, comparison of combined CoB and dual adhesion blockade to CoB alone yielded p<.0001. Comparison between MHV68-infected animals treated with CoB alone, CoB+anti-LFA-1, and CoB+anti-VLA-4 revealed no significant differences (p = 0.103). C) Kaplan-Meier survival curves comparing skin graft survival between the following groups: Non-infected mice treated with CoB (solid black line, MST 22 d, n = 48, 8 independent experiments); Non-infected mice treated with CoB+anti-LFA-1/anti-VLA-4 (dotted black line, MST>100 d, n = 26, 3 independent experiments); MHV68-infected mice treated with CoB (solid red line, MST 13.5, n = 40, 7 independent experiments); MHV68-infected mice treated with CoB+anti-LFA1/anti-VLA4 (solid green line, MST>100 d, n = 25, 3 independent experiments). Statistical comparisons of skin graft survival between groups were made with the log-rank test. In non-infected animals, comparison of combined CoB+dual adhesion blockade to CoB alone resulted in p<.0001. In MHV68-infected animals, comparison of CoB+anti-LFA-1/anti-VLA-4 treatment to CoB treatment yielded p<.0001. (D) Maintenance adhesion blockade (anti-LFA1/anti-VLA4) therapy was discontinued from 8 MHV68-infected mice (MHV68 WT or MHV68 M1.STOP) treated with CoB+anti-LFA1/anti-VLA4 and who had stable surviving allografts after 90-100 days of treatment with dual adhesion blockade. Following discontinuation of therapy, all grafts failed with a median time of 34 d. (E) Mice treated with combined costimulation and adhesion blockade demonstrated a decreased frequency of KLRG1+/CD127+ CD8+ T cells relative to mice treated with costimulation blockade alone. Gray columns: MHV68-infected mice treated with CoB. Red columns: MHV68-infected mice treated with CoB+antiLFA1/anti-VLA4. Y axis: % of KLRG1+/CD127+ CD8+ T cells expressed as a percentage of total CD8+ T cells. After accounting for multiple testing using the Holm method, this effect was significant at 28 days after skin grafting. In mice treated with CoB alone (n = 6), 33.3% of CD8+ T cells were KLRG1+/CD127+ versus 19.9% in mice with combined treatment (n = 4, p = .042*). Error bars represent the standard error of the mean.

Given the upregulation of both LFA-1 and VLA-4 that we observed on the CD8^dim^ cells that expanded during viral latency, the contributions of these two adhesion molecules to graft survival, and their ability to combine with CoB, were tested. As shown in Figure 6A, combined blockade of LFA-1 and VLA-4 (without CoB) produced a statistically significant prolongation in skin graft survival in infected mice compared to CoB alone (MST 27 d with adhesion blockade, n = 14, p = .002 compared to CoB alone), resulting in graft survival in MHV68-infected animals that was indistinguishable from that observed in the non-infected cohort treated with either CoB alone or anti-LFA-1/anti-VLA-4 alone (MST 22 d for CoB treatment and 24 d for anti-LFA-1/anti-VLA-4 in uninfected animals, p = n.s.). Of note, in the non-infected cohort, blockade of LFA-1 and VLA-4 alone (without CoB) did not prolong graft survival in comparison to CoB alone (MST 22 d for CoB treatment and 24 d for anti-LFA-1/anti-VLA-4, respectively, p = n.s.).

While treatment with anti-LFA-1 and anti-VLA-4 adhesion blockade without concomitant CoB modestly prolonged allograft survival in MHV68-infected animals, as shown in Figure 6B, when both adhesion blockade and CoB were combined, allograft survival was greatly prolonged. Thus, treatment with CoB+anti-LFA-1/anti-VLA-4 resulted in an MST of >100 d in MHV68-infected animals (n = 25), p<.0001 compared to CoB alone, and p<.0001 compared to adhesion blockade alone (Figure 6B). Importantly, as shown in Figure 6C, the combination of CoB+adhesion blockade resulted in graft prolongation for both non-infected and infected animals (MST>100 d for both cohorts) suggesting that the mechanism of CoBRR includes adhesion pathways in both non-infected and infected settings. While both the non-infected and the infected cohorts demonstrated MSTs of >100 d when treated with CoB+adhesion blockade, the non-infected cohort demonstrated higher rates of allograft survival at the 100 day experimental end-point compared to the infected cohort (100% of grafts surviving in non-infected animals versus 68% of grafts surviving in MHV68-infected animals, p = .002, Figure 6C). This data suggests that additional pathways (resistant to both CoB and adhesion blockade) may be functioning in the MHV68-infected recipients, and that these can lead to an increased risk of allograft rejection despite combined CoB+adhesion blockade. This is consistent with the phenotypic analysis shown in Figures 2, 3, and 4 which demonstrate multimodal signs of immune deviation in MHV68-infected animals.

To determine whether blockade of either LFA-1 or VLA-4 played a predominant role in graft acceptance, graft recipients were treated with CoB along with either anti-LFA-1 or anti-VLA-4 as single anti-adhesion therapies. In MHV68-infected animals, the addition of adhesion blockade monotherapy did not significantly prolong skin graft survival (CoB+anti-LFA-1, MST 16 d, n = 12; CoB+anti-VLA-4 MST 20 d, n = 11; p = .10 compared to CoB alone Figure 6B). This is in contrast to observations previously published by our research group, which showed that in non-infected animals, treatment with anti-LFA-1 alone (no anti-VLA-4) combined with CTLA-4-Ig significantly prolonged skin graft survival [23].

As shown in Figure 6D, the impact of adhesion blockade on graft rejection required ongoing treatment with anti-LFA-1 and anti-VLA-4. When adhesion blockade was withdrawn from eight virally-infected recipients with ongoing graft survival at 100 days post-transplant, prompt rejection occurred (median of 34 d after adhesion blockade withdrawal, Figure 6D).

Consistent with the salutary effect of anti-LFA-1/anti-VLA-4 adhesion blockade on graft acceptance, the addition of this blockade also partially normalized the T cell phenotype observed in the blood of infected animals. Thus, as shown in Figure 6E, we observed a decreased proportion of CD127+/KLRG1+ CD8+ T cells in infected animals treated with combined costimulation and adhesion blockade relative to animals treated with CoB alone.

**Figure 7:**
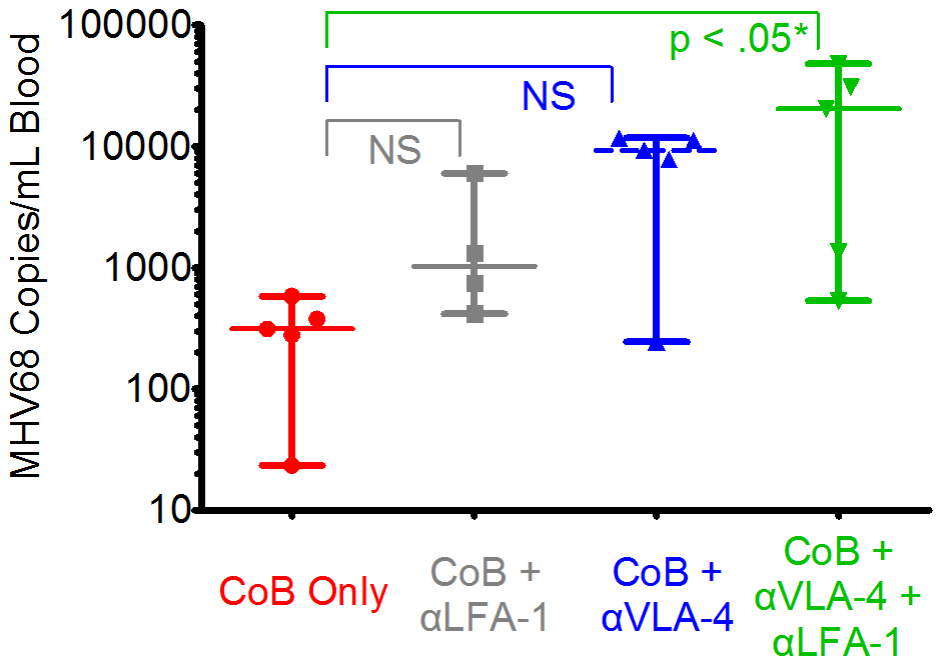
The addition of adhesion blockade to costimulation blockade increases viral replication. MHV68 viral loads were measured>100 days after treatment with CoB +/− adhesion blockade by PCR for MHV68 ORF50 [19]. Recipients treated with CoB alone (red data points) showed a median value of 313 copies/mL (n = 5). Recipients treated with CoB+anti-LFA-1 alone (gray data points) showed a median value of 1027/mL (n = 4, p = n.s. compared to CoB alone). Recipients treated with CoB+anti-VLA-4 alone (blue data points) showed a median value of 9255/mL (n = 5, p = n.s. compared to CoB alone). Recipients treated with CoB+anti-LFA-1+ anti-VLA-4 (green data points) showed a median value of 20278/mL (n = 5, p<.05 compared to CoB alone). Plots for each group show the median viral load with range from a single experiment.

Given that previous studies have documented defects in anti-viral protective immunity with adhesion blockade [47], [48], plasma MHV68 loads between treatment groups were compared using a quantitative PCR assay (Figure 7), to determine if the risk of MHV68 reactivation would increase with combination therapy. This analysis showed that combined adhesion blockade led to increased viral loads. Thus, in mice treated with CoB alone, the median viral load (measured>100 days post-transplant) was 313 copies/mL (range: 23–581, n = 5, Figure 7). The addition of weekly anti-LFA-1 treatment to CoB induction resulted in a modestly increased viral load (median: 1027, range: 417–5974, n = 4, Figure 7). Weekly anti-VLA-4 treatment with CoB induction increased viral loads to a median of 9255 (range: 244–11685, n = 5, Figure 7) and combined weekly anti-LFA-1 and anti-VLA-4 with CoB induction resulted in the highest viral loads (median: 20278, range: 532–47663, p<.05 compared to CoB alone, n = 5, Figure 7).

**Figure 8:**
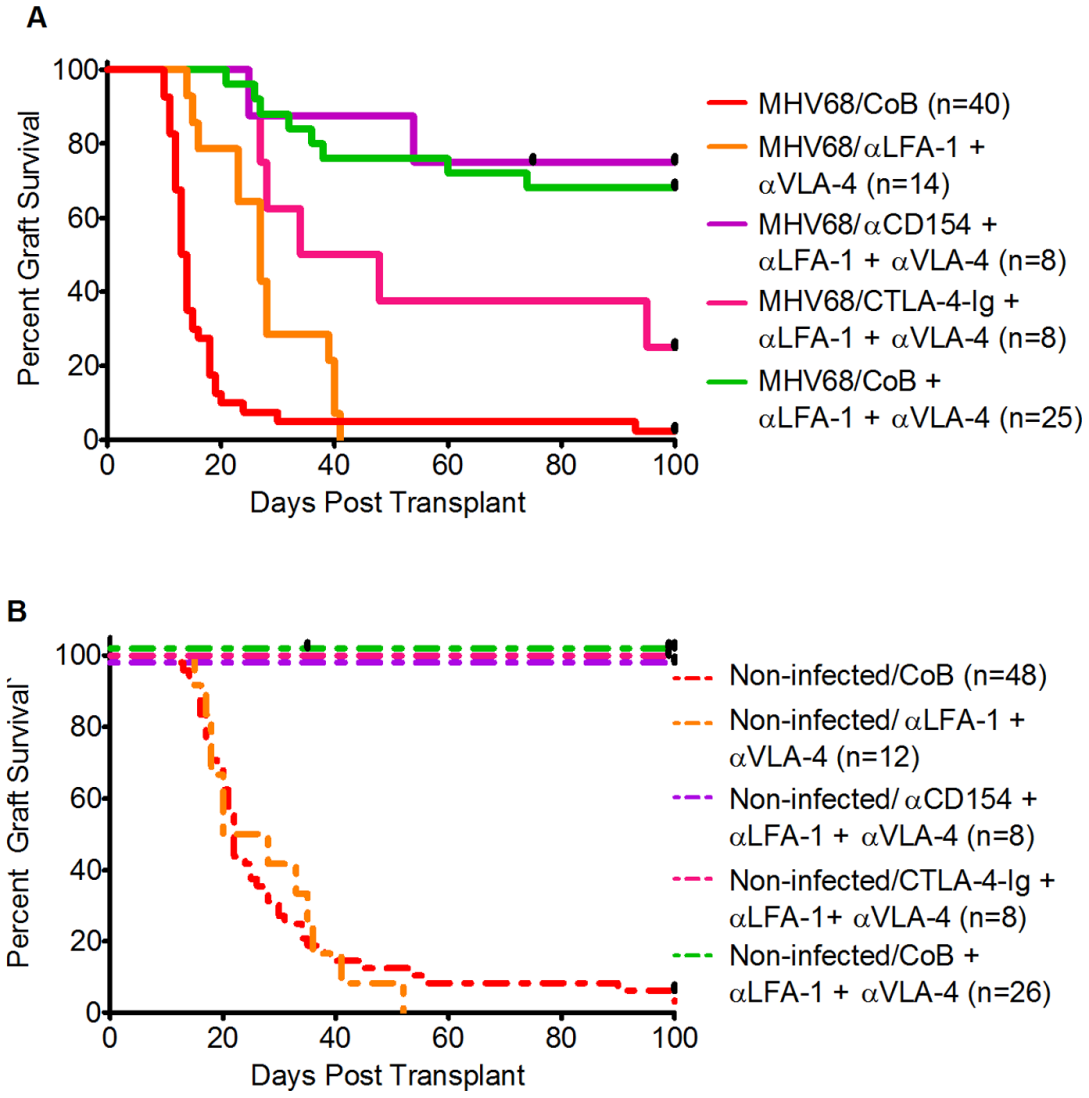
Comparative Effect of CTLA-4-Ig and anti-CD154+ adhesion blockade in MHV68-infected and non-infected mice. A) Kaplan-Meier survival curves comparing skin graft survival between the following groups of MHV68-infected mice: MHV68-infected mice treated with CoB (solid red line, MST 13.5 d, n = 40, 7 independent experiments); MHV68-infected mice treated with anti-LFA-1+ anti-VLA-4 (solid orange line, MST 27 d, n = 14, 2 independent experiments); MHV68-infected mice treated with anti-CD154+ anti-LFA-1/anti-VLA-4 (solid violet line, MST>100 d, n = 8, one experiment); MHV68-infected mice treated with CTLA-4-Ig+anti-LFA-1/anti-VLA-4 (solid magenta line, MST 41 d, n = 8, one experiment); MHV68-infected mice treated with CoB+anti-LFA-1/anti-VLA-4 (solid green line, MST>100 d, n = 25, 3 independent experiments). Skin graft survival between groups was compared using the log-rank test. In infected animals, the combination of anti-LFA-1 and anti-VLA-4 was compared to CoB alone resulting in p = .0023. CTLA-4-Ig+anti-LFA-1/anti-VLA-4 compared to CoB alone yielded p = .0002. The combination of anti-CD154+ anti-LFA-1/anti-VLA-4 was significantly different from CoB alone (p<.0001) and not significantly different from dual CoB (anti-CD154+ CTLA-4-Ig)+dual adhesion blockade (p = .73). B) Kaplan-Meier survival curves comparing skin graft survival between the following groups of non-infected mice: non-infected mice treated with CoB (dotted red line, MST 22 d, n = 48, 8 independent experiments); non-infected mice treated with anti-LFA-1+ anti-VLA-4 (dotted orange line, MST 24, n = 12, 2 independent experiments); non-infected mice treated with anti-CD154+ anti-LFA-1/anti-VLA-4 (dotted violet line, MST>100 d, n = 8, one experiment); non-infected mice treated with CTLA-4-Ig+anti-LFA-1/anti-VLA-4 (dotted magenta line, MST>100 d, n = 8, one experiment); non-infected mice treated with CoB+anti-LFA-1/anti-VLA-4 (dotted green line, MST>100 d, n = 26, 3 independent experiments). Graft survival between groups in non-infected animals was compared with the log-rank method. Survival in the CoB only and anti-LFA-1+ anti-VLA-4 groups was not significantly different (p = .87). Relative to CoB alone, survival was significantly prolonged in non-infected animals treated with anti-CD154+ anti-LFA-1/anti-VLA-4 (p<.0001), CTLA-4-Ig+anti-LFA-1/anti-VLA-4 (p<.0001), or dual CoB (anti-CD154+ CTLA-4-Ig)+anti-LFA-1/anti-VLA-4 (p<.0001).

Given the reciprocal balance that was observed between graft acceptance and MHV68 viral load when combined CTLA-4-Ig+anti-CD154 CoB and adhesion blockade were simultaneously employed, we next determined whether a more limited immunomodulatory platform could be designed that was still capable of overcoming MHV68-mediated allograft rejection. As shown in Figure 8A, when CTLA-4-Ig alone (without anti-CD154) was combined with dual adhesion blockade in MHV68-infected animals, allograft survival was significantly shortened compared to when adhesion blockade was combined with both CTLA-4-Ig and anti-CD154 (MST 41 d with CTLA-4-Ig alone and adhesion blockade, p = .024 compared to MST>100 d with combined CoB+adhesion blockade). In contrast, the combination of anti-CD154 (without CTLA-4-Ig) and dual adhesion blockade in infected animals was similarly effective to combined costimulation- and adhesion- blockade in prolonging allograft acceptance. (MST>100 d, p = n.s. compared to combined CoB+adhesion blockade, Figure 8A).

The results with single CoB+adhesion blockade in MHV68-infected animals were distinct from what was observed in non-infected recipients, where significant prolongation of allograft survival occurred when either CTLA-4-Ig alone or anti-CD154 alone was paired with dual adhesion blockade (MST>100 d for both groups, Figure 8B). These results underscore the immunologic distinctions between non-infected and infected recipients, and suggest that the increased risk of rejection that MHV68-induces may derive at least in part from a virus-associated escape from the normal requirement for CD28/B7 signaling for allograft rejection.

## Discussion

As costimulation blockade-based therapies are increasingly used for immunosuppression during solid organ transplantation, it is critical to understand the interactions that occur between this new class of agents and the T cell repertoire that is shaped by both antecedent and ongoing viral infections. Given that the majority of transplant patients harbor several latent herpesviruses, the impact of these viruses in their latent state on immunity and rejection is of paramount clinical importance. While previous work has concentrated on either acute infections or transgenic systems in which antigen cross-reactivity is modeled [1], [6], [8], here we have developed a system to study the impact of a clinically relevant, single, latent infection on alloimmunity. We have found that latent MHV68 infection has a profound impact on the activation state of the recipient immune system, leading to changes in cytokine secretion, and to changes in the phenotype of CD8+ T cells. These immunologic deviations correlated with enhanced transplant rejection in MHV68-infected animals, despite T cell CoB; MHV68 infection lead to a statistically and clinically significant reduction in the MST of BALB/c skin grafts placed onto B6 recipients treated with CoB, reducing graft acceptance to what has been previously observed in non-infected animals given no peri-transplant immunosuppression (Figure 1 and [22], [23]). The absolute reduction in MST attributable to MHV68 infection represented near-total abrogation of the effect of CoB in the BALB/c to B6 skin graft model.

The complexity and severity of the immune response to latent MHV68 infection was demonstrated by the increase in plasma chemokines, the large expansion of CD8^dim^ T cells, and the phenotypic alterations that were observed in these cells in the setting of MHV68 latency. The increased secretion of CXCL9 and CXCL10 is notable, given the critical roles that these molecules may play in both alloreactivity and protective immunity: While the receptor for these ligands, CXCR3, plays a role in protective immunity against MHV68 [49], this axis has also been implicated in the pathogenicity of alloimmunity to murine skin, cardiac, and islet allografts as well as human lung and renal transplants [25]-[27], [50]. We additionally found a correlation of the expansion of a CD8^dim^ T cell subpopulation with latent MHV68 viral infection. Many of these cells were characterized by the CD127+/KLRG1+ double-positive T_EM_ [51] phenotype, that was moderately attenuated during graft prolongation with combined CoB and adhesion blockade (Figure 6C). This is of particular interest, given multiple previous studies that have strongly associated T_EM_ wth CoBRR. [13], [14], [52]-[54]. CD8^dim^ cells were also characterized by their decreased expression of multiple costimulation markers and of increased expression of the adhesion proteins LFA-1 and VLA-4. These findings highlight the complexity of the potential mechanisms for heterologous alloimmunity, which may include both soluble and cell-mediated mediators of immune activation and subsequent rejection. While our decision to use adhesion blockade to overcome virus-induced CoBRR was influenced by the increased expression of LFA-1 and VLA-4 on CD8^dim^ T cells, these observations are not sufficient to prove causality of the CD8^dim^ cells in transplant rejection. Direct evaluation of the role of CD8^dim^ in MHV68-mediated CoBRR would require evidence of allograft rejection following adoptive transfer of CD8^dim^ into immunodeficient mice. Unfortunately, several technical issues preclude such experiments at present: Individual CD8^dim^ appear to be transient in nature and without ongoing viral replication as a stimulus, adoptively transferred CD8^dim^ are unlikely to persist. In addition, MHV68 is known to establish latency in several peripheral immunologic cell lines [55]. Adoptive transfer of CD8^dim^ thus may inadvertently transfer virus making it difficult to distinguish the effects of CD8^dim^ from direct effects of the virus. Finally, preliminary experiments suggested that as many as 200×10^6^ CD8^dim^ cells would need to be transferred to each recipient to recapitulate the expansion of these cells observed in the setting of MHV68 infection, which is not a feasible cell dose to give individual mice. Given these limitations, it is not possible to attribute a causative link between the expansion of CD8^dim^ cells and latency-induced rejection. However, the indirect evidence of a role for CD8^dim^ presented here suggests that further study of this unique cell population is warranted. Indeed, the expansion of a phenotypically similar population has been observed in the setting of persistent viral infection in several clinical scenarios including after infection with either HIV or EBV [56], [57]. Further study of these cells in both mouse models and in patients will be required to uncover any mechanistic links to immune dysregulation.

As noted above, the fact that the MHV68-associated CD8^dim^ population demonstrated decreases in costimulatory molecule expression (Figure 4A), and increased expression of LFA-1 and VLA-4 (Figure 4B) suggested that adjunctive blockade of these two molecules might overcome the heterologous immune barrier induced by MHV68. Indeed, as was demonstrated in Figure 6A, treating infected recipients with adhesion blockade alone (without CoB) significantly increased the duration of skin graft survival compared to CoB alone, leading to skin allograft MSTs that were indistinguishable from that observed in non-infected animals treated with adhesion blockade. Recent evidence suggests that the prevailing effect of VLA-4 blockade when combined with CoB in transplantation is impaired trafficking of activated memory T cells into the transplanted tissue [8]. Similarly, LFA-1 blockade with CoB decreases infiltration of memory T cells into the graft, but additionally appears to impair memory T cell effector function [6], [8]. Thus, the salutary effects of combined LFA-1 and VLA-4 blockade may have derived from their interference with T cell trafficking, their impairment of alternative costimulation through LFA-1 or VLA-4, impaired effector function through blockade of LFA-1, or a combination of these effects [6], [8], [58]. The importance of the block in trafficking and effector function was underscored by the need for ongoing adhesion blockade to assure graft acceptance in our system: following withdraw of LFA-1 and VLA-4 blockade, long-surviving allografts that were placed in the setting of combined CoB+adhesion blockade were promptly rejected, possibly due to previously restrained T cells now able to traffic to the allograft and promote rejection. This observation may be important clinically, where there may be a rebound effect of increased alloreactivity in the setting of discontinuation of adhesion blockade used during induction immunosuppression regimens.

While dual adhesion blockade alone was capable of moderately lengthening skin graft survival compared to CoB in MHV68-infected animals, to achieve prolonged allograft acceptance, combination treatment with both CoB+dual anti-LFA1/anti-VLA4 adhesion blockade was required. In contrast to the present studies, previous experiments conducted in the setting of heterologous alloimmunity induced by sequential acute infections [6] or in non-infected animals [23] required only single-pathway adhesion blockade (with anti-LFA-1) in combination with CoB to overcome transplant rejection. This observation suggests that the continual presence of latent virus may increase the alloimmune barrier and underscores the potential importance of using latent virus models of heterologous immunity in preclinical studies, in order to more faithfully recapitulate the immune milieu that predominates in transplant recipients. Thus, the model described herein provides a platform to further study heterologous alloimmunity in a clinically-relevant model.

The observation that adhesion blockade complements CoB in the setting of latent-virus-induced heterologous alloimmunity provides mechanistic insight, but also highlights the ever-present challenge in transplantation of balancing the benefits of immunosuppression against its impact on protective immunity. As demonstrated in this study (Figure 7), combined costimulation and adhesion blockade resulted in significant increases in MHV68 viral load. This observation highlights the complex interactions that are expected between effective control of the allo- and viral-specific immune responses, and suggests that work to define the minimal successful regimen for graft prolongation in the setting of viral latency may be critical to ensure the most successful balance between transplant acceptance and preservation of protective immunity. In this study, we have shown that in MHV68-infected animals, single CoB with anti-CD154 (without CTLA-4-Ig) could effectively replace dual CoB when combined with anti-LFA-1/anti-VLA-4 adhesion blockade (Figure 8A). This observation not only suggests a potentially safer strategy to overcome latent virus-induced alloreactivity, but also further distinguishes the immune barriers that exist in infected versus non-infected animals. Moreover, the ineffectiveness of CTLA-4-Ig in the setting of latent viral infection may have significant clinical ramifications, given that most transplant patients harbor latent herpesviruses, This data suggests that development of safe strategies to target the CD154 pathway may provide significant benefit to patients undergoing transplantation in the setting of the complex immune consequences that exist in the setting of latent viral infections.

## Supporting Information

Table S1 **List of cytokines and chemokines tested in multiplex assay.** These cytokines and chemokines are included in the premixed 32-plex Millipore MILLIPLEX MAP Cytokine/Chemokine kit. These analytes were assessed every two weeks for ten weeks following infection of C57BL/6 (B6) mice with MHV68 and levels compared to those in non-infected mice.
(DOC)

Table S2 **Summary of qPCR gene expression data comparing CD8 subtypes.** All transcripts that were found to be significantly different between the three groups of CD8 T cells (non-infected, infected-CD8^dim^, infected-CD8^bright^) are included in this table. Additionally, relative expression and the results of statistical testing are shown for pairwise comparison between groups. P-values marked with an asterisk* indicate genes for the CD8-pair that had a difference in expression of at least two-fold and were statistically-significantly different.
(DOCX)
