# Supplementary material for: Heterologous Immunity Triggered by a Single, Latent Virus in Mus musculus: Combined Costimulation- and Adhesion- Blockade Decrease Rejection
Source: PLoS One. 2013 Aug 5;8(8):e71221. doi: 10.1371/journal.pone.0071221 (PMC3733932; doi:10.1371/journal.pone.0071221)
Supplement: Table S1 — List of cytokines and chemokines tested in multiplex assay. These cytokines and chemokines are included in the premixed 32-plex Millipore MILLIPLEX MAP Cytokine/Chemokine kit. These analytes were assessed every two weeks for ten weeks following infection of C57BL/6 (B6) mice with MHV68 and levels compared to those in non-infected mice. (DOC) [file pone.0071221.s001.doc]

| Eotaxin | IL-6 |
| --- | --- |
| G-CSF | IL-7 |
| GM-CSF | IL-9 |
| IFN-γ | IP-10 |
| IL-10 | KC |
| IL-12 (p40) | LIF |
| IL-12 (p70) | LIX |
| IL-13 | M-CSF |
| IL-15 | MCP-1 |
| IL-17 | MIG |
| IL-1α | MIP-1α |
| IL-1β | MIP-1β |
| IL-2 | MIP-2 |
| IL-3 | RANTES |
| IL-4 | TNF-α |
| IL-5 | VEGF |
