# Supplementary material for: Heterologous Immunity Triggered by a Single, Latent Virus in Mus musculus: Combined Costimulation- and Adhesion- Blockade Decrease Rejection
Source: PLoS One. 2013 Aug 5;8(8):e71221. doi: 10.1371/journal.pone.0071221 (PMC3733932; doi:10.1371/journal.pone.0071221)
Supplement: Table S2 — Summary of qPCR gene expression data comparing CD8 subtypes. All transcripts that were found to be significantly different between the three groups of CD8 T cells (non-infected, infected-CD8dim, infected-CD8bright) are included in this table. Additionally, relative expression and the results of statistical testing are shown for pairwise comparison between groups. P-values marked with an asterisk* indicate genes for the CD8-pair that had a difference in expression of at least two-fold and were statistically-significantly different. (DOCX) [file pone.0071221.s002.docx]

|  | | **CD8^dim^ vs CD8^non^** | | | **CD8^bright^ vs CD8^non^** | | | **CD8^dim^ vs CD8^bright^** | | |
| --- | --- | --- | --- | --- | --- | --- | --- | --- | --- | --- |
| *Analyte* | *p for comparison of all groups* | *Relative Expression (CD8dim vs CD8non)* | *Relative Expression ± 1 SD (Range)* | *p for CD8dim vs CD8non comparison* | *Relative Expression (CD8bright vs CD8non)* | *Relative Expression ± 1 SD (Range)* | *p for CD8bright vs CD8non comparison* | *Relative Expression (CD8dim vs CD8bright)* | *Relative Expression ± 1 SD (Range)* | *p for CD8dim vs CD8bright comparison* |
| Bcl2 | 0.0447 | 0.32 | (0.24 - 0.42) | 0.029* | 0.60 | (0.38 - 0.95) | 0.491 | 0.53024 | (0.33 - 0.85) | 0.105 |
| Ccl3 | 0.0343 | 18.40 | (11.26 - 30.07) | 0.029* | 11.13 | (6.08 - 20.38) | 0.018* | 1.65297 | (0.86 - 3.16) | 0.220 |
| Ccl5 | 0.0343 | 14.22 | (9.92 - 20.39) | 0.029* | 6.20 | (4.38 - 8.78) | 0.018* | 2.29317 | (1.42 - 3.71) | 0.024* |
| Ccr2 | 0.0343 | 12.29 | (7.63 - 19.79) | 0.029* | 5.68 | (3.93 - 8.23) | 0.018* | 2.16245 | (1.32 - 3.54) | 0.024* |
| Ccr7 | 0.0343 | 0.03 | (0.03 - 0.04) | 0.029* | 0.66 | (0.3 - 1.46) | 0.945 | 0.05155 | (0.02 - 0.12) | 0.004* |
| Cd38 | 0.0343 | 3.41 | (2.38 - 4.87) | 0.029* | 2.48 | (1.6 - 3.85) | 0.018* | 1.37112 | (0.84 - 2.23) | 0.220 |
| Cd80 | 0.0447 | 6.02 | (3.12 - 11.65) | 0.029* | 3.47 | (1.68 - 7.17) | 0.073 | 1.73754 | (0.8 - 3.76) | 0.413 |
| Fasl | 0.0343 | 14.80 | (9.33 - 23.46) | 0.071 | 7.05 | (3.91 - 12.74) | 0.050* | 2.0973 | (1.04 - 4.22) | 0.066 |
| Gzmb | 0.0447 | 28.85 | (20.64 - 40.34) | 0.029* | 27.71 | (14.82 - 51.81) | 0.018* | 1.04118 | (0.53 - 2.03) | 1 |
| H2-Eb1 | 0.0447 | 0.17 | (0.11 - 0.28) | 0.029* | 0.45 | (0.23 - 0.89) | 0.691 | 0.38363 | (0.18 - 0.81) | 0.105 |
| Hmox1 | 0.0447 | 0.19 | (0.12 - 0.31) | 0.029* | 0.14 | (0.09 - 0.24) | 0.048* | 1.35726 | (0.77 - 2.41) | 1 |
| Prf1 | 0.0343 | 4.07 | (2.52 - 6.6) | 0.029* | 2.35 | (1.5 - 3.67) | 0.036* | 1.73292 | (0.96 - 3.12) | 0.220 |
| CD45 | 0.0343 | 4.36 | (2.69 - 7.05) | 0.029* | 3.00 | (2.36 - 3.81) | 0.018* | 1.45306 | (0.86 - 2.46) | 0.105 |
| Stat1 | 0.0406 | 1.27 | (0.8 - 2.03) | 1 | 2.32 | (1.66 - 3.24) | 0.018* | 0.54882 | (0.32 - 0.93) | 0.042* |
| T-bet | 0.0343 | 4.63 | (3.12 - 6.86) | 0.029* | 2.74 | (1.84 - 4.07) | 0.018* | 1.68894 | (1.05 - 2.72) | 0.154 |
